# Learning Attention Representation with a Multi-Scale CNN for Gear Fault Diagnosis under Different Working Conditions

**DOI:** 10.3390/s20041233

**Published:** 2020-02-24

**Authors:** Yong Yao, Sen Zhang, Suixian Yang, Gui Gui

**Affiliations:** 1 School of Mechanical Engineering, Sichuan University, Chengdu 610065, China; 2 University of Chinese Academy of Sciences, Beijing 100049, China; 3 Chengdu Institute of Computer Application, Chinese Academy of Sciences, Chengdu 610041, China; 4 National institute of Measurement and Testing Technology, Chengdu 610021, China

**Keywords:** acoustic-based diagnosis, gear fault diagnosis, attention mechanism, convolutional neural network

## Abstract

The gear fault signal under different working conditions is non-linear and non-stationary, which makes it difficult to distinguish faulty signals from normal signals. Currently, gear fault diagnosis under different working conditions is mainly based on vibration signals. However, vibration signal acquisition is limited by its requirement for contact measurement, while vibration signal analysis methods relies heavily on diagnostic expertise and prior knowledge of signal processing technology. To solve this problem, a novel acoustic-based diagnosis (ABD) method for gear fault diagnosis under different working conditions based on a multi-scale convolutional learning structure and attention mechanism is proposed in this paper. The multi-scale convolutional learning structure was designed to automatically mine multiple scale features using different filter banks from raw acoustic signals. Subsequently, the novel attention mechanism, which was based on a multi-scale convolutional learning structure, was established to adaptively allow the multi-scale network to focus on relevant fault pattern information under different working conditions. Finally, a stacked convolutional neural network (CNN) model was proposed to detect the fault mode of gears. The experimental results show that our method achieved much better performance in acoustic based gear fault diagnosis under different working conditions compared with a standard CNN model (without an attention mechanism), an end-to-end CNN model based on time and frequency domain signals, and other traditional fault diagnosis methods involving feature engineering.

## 1. Introduction

As a one of the most important components in transmission systems, gears are widely used in many types of machinery, such as wind turbines, construction machinery, automobiles, and other fields [1], thanks to their unique merits, such as large transmission ratio, high efficiency, and heavy load capacity [2,3]. The working performance of gears directly influences the operational reliability of the whole machinery [4]. However, due to poor environmental conditions and the intensive impact load operational condition of transmission systems, gears are vulnerable to display some faults and cause the machine to break down. This may lead to significant economic losses [5]. Therefore, research on fault diagnosis for gears can effectively avoid catastrophic failure and reduce economic loss.

Recently, the fault diagnosis of gears has been extensively studied by researchers. However, most current studies focus on mainly stable working conditions. In the real world, gears usually work under variable and fluctuant operation conditions [6]. As such, the nonlinear and non-stationary characteristics of signals under variable conditions exhibit many unique characteristics, such as strong nonstationary, frequency mixing, and modulated phenomena [7]. Traditional fault diagnosis methods and technologies, which are only applicable to gears under stationary conditions [1], are incapable of detecting and identifying gear fault patterns under variable conditions.

To solve this issue, gear fault diagnosis under variable conditions has become the subject of extensive research and has aroused researchers’ great concern in the past few years. Liu et al. [8] proposed a method for gear fault diagnosis under slight variations in working conditions via empirical mode decomposition (EMD) and multi-fractal detrended cross-correlation analysis (MFDCCA). By using EMD and MFDCCA methods, the multi-fractal fault features can effectively extract and distinguish fault modes. In order to avoid mode mixing, Chen et al. [9] proposed to use complementary ensemble empirical mode decomposition (CEEMD) technology to decompose the raw vibration signals and select the intrinsic mode functions (IMFs) using a correlation analysis algorithm (CorAA) for a probabilistic neural network to classify the gear fault patterns under different working conditions. Xing et al. [10] adopted the intrinsic time-scale decomposition (ITD) and singular value decomposition methods to improve the robustness of gear fault feature extraction under variable conditions. Zhang et al. [11] proposed a method for gear fault diagnosis under different working conditions based on local characteristic-scale decomposition (LCD) denoising and the vector mutual information method. Chen et al. [12] performed gearbox fault diagnosis under variable speed conditions via analysis of the torsional vibration signals in the time-frequency domain. Though these studies, rich methods, and technologies for gear fault diagnosis have been accumulated and provide a pivotal function under variable conditions, most of the methods typically use vibration signals as the main measurement values to diagnose gear faults in variable working conditions for use in vibration analysis [13]. In many practical conditions, the installation of vibration sensors is constrained by some working conditions and the complex structure of the equipment themselves, which makes the signal acquisition inconvenient. Moreover, vibration signals are easily masked in some special environments, such as high humidity, high temperature, and high corrosion; therefore, the application of vibration signal analysis methods for gear fault diagnosis under variable conditions is limited due to the requirement of contacted measuring. Meanwhile, those studies that adopt vibration analysis methods, usually rely on signal processing technology to decompose raw vibration signals into several proper signal components to extract valuable features for distinguishing gear fault patterns under different working conditions. Although all these vibration signal analysis methods can work well in fault mode detection tasks, they rely heavily on diagnostic expertise and prior knowledge of signal processing technology [14], which may lead to tedious and inefficient procedures in practical diagnosis tasks. Considering the existing issues, the effective methods and technologies of gear fault diagnosis under variable conditions still needs to be further developed.

As a typical non-contact measurement, acoustic-based diagnosis (ABD) methods, which have the capability to overcome the limitation of vibration measurement, are widely used in the fault diagnosis field. Lu et al. [15,16,17] proposed an acoustic-based fault diagnosis method based on near-field acoustic holography for detecting gear fault patterns under stationary working conditions. Glowacz [18,19] design several acoustic-based diagnosis methods with novelty acoustic features to detect the fault of commutator motors, electric impact drills, and coffee grinders. By combing time-frequency data fusion technology and the Doppler feature matching search (DFMS) algorithm, Zhang et al. [20] proposed a train bearings fault diagnosis method, which is based on wayside acoustic signals. Inspired by their study, Zhang et al. [21] designed an improved singular value decomposition with a resonance-based wayside acoustic signal sparse decomposition technique as an adaptive form of train bearings fault feature extraction. However, like the vibration-based diagnosis method, all the existing acoustic-based methods are also heavily rely on prior knowledge of signal processing technology rather than utilizing intelligent fault diagnosis techniques. This is because the fault data distribution that we obtain in one working condition are not consistent in another different working condition in real applications [22], which means the distribution difference between training data and test data changes as the working condition varies, which can lead to a dramatic drop in performance.

To manage the obstacles, we considered the role of the attention mechanism. As a novel intelligent method, the attention mechanism, which has the capability to adaptively capture temporal correlations between different sequences [23] and allows for feature extraction networks to focus on the relevant characteristics without signal processing technology and feature engineering, are commonly explored in various structural prediction tasks, such as document classification [24], speech recognition [25,26,27], and environmental classification [28,29]. Therefore, in this paper, we propose a novel ABD method for gear fault diagnosis under different working conditions based on a multi-scale convolutional learning structure and attention mechanism. In our methods, a multi-scale convolutional learning structure was designed to automatically mine multi-scale features using different filter banks from raw acoustic signals. Then, a novel attention mechanism, which was based on a multi-scale convolutional learning structure, was established to adaptively allow the multi-scale network to focus on relevant fault pattern information under different working conditions. Finally, a stacked convolutional neural network (CNN) model was proposed to detect the fault mode of the gears.

The main contributions of this paper are as follows:

1. We are the first to propose an acoustic-based diagnosis method to detect the fault patterns under different working conditions, where this method obtains information directly from raw acoustical signals without manual signal processing and feature engineering.
2. We are the first to introduce the attention mechanism theory into the acoustic-based diagnosis field to address gear fault pattern recognition under different working conditions by designing a novel attention-based mechanism that is based on a multi-scale convolutional learning structure to adaptively extract relevant fault patterns information and reduce data distribution variation under different working conditions.
3. We designed a novel attention-based, multi-scale CNN model based on the two innovations above. It outperformed a single-scale network and multi-scale network without attention mechanism, and achieved favorable results relative to other methods using manual feature engineering based on the function of multi-scale structure and an attention mechanism.

## 2. Model Building

**Figure 1:**
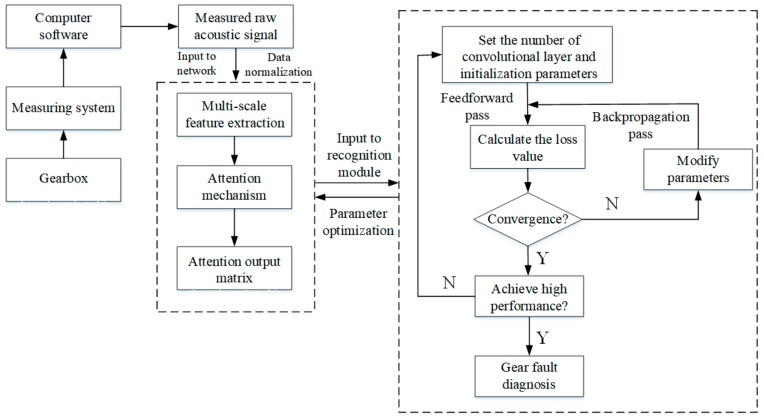
Block diagram of the proposed method.

In this section, we briefly introduce the mathematical model of our acoustic-based gear fault diagnosis method, which can be roughly divided into three parts. In the first part, a multi-scale convolutional layer operates directly on raw acoustic signals and automatically mines fault features using different filter sizes and strides to construct feature vectors. In the second part, an attention mechanism is adopted to obtain reasonable attention weight vectors from the convolutional layer, which are multiplied with each feature vector of the pooling layer. In the last part, the multi-dimensional attention output matrix, which is concatenated with the multi-scale attention structure, is constructed as a stacked CNN input to train the network. The block diagram of the proposed method is shown in Figure 1.

### 2.1. Multi-Scale Convolution Operation

A commonly used approach for an end-to-end neural network is to pass the raw acoustic signal through a 1D convolutional layer, which has a fixed filter size and stride length to create invariance to phase shifts and further down-sample the signals. However, those methods are still constrained in various prediction tasks for two reasons: (1) There is always a trade-off when choosing the filter size. A high-scale filter size may have a good frequency resolution but does not have a sufficient filter for location in the high frequency area. A low-scale filter size, on the contrary, focuses on more frequency bands but has a low resolution [30,31]. (2) Features extracted using a fixed filter size cannot make full use of the raw signal information to build a discriminative representation for different patterns. Considering this, a multi-scale convolutional function, which has the capability to learn discrepant features, has been applied to address the obstacles. By extracting features with multiple different scale filter banks and splitting responsibilities based on what filter banks can efficiently represent, multi-scale convolutions have already been successfully used in various recognition fields, such as image classification [32], environmental sound classification [30], and speech recognition [33].

**Figure 2:**
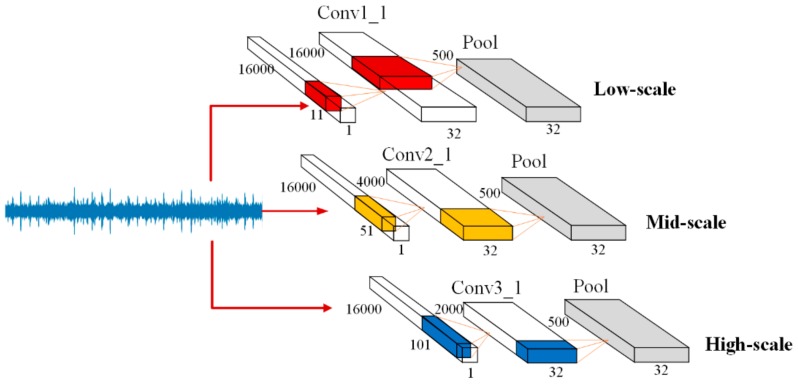
Multi-scale feature extraction mechanism.

Inspired by their work, we designed a multi-scale convolutional learning structure to extract multi-scale fault features from raw acoustic signals. The structure is shown in Figure 2.

Three scale convolution kernels were used to operate the input signal vector to extract different features and increase the bias to achieve a result. The function is defined as followed:

(1) $$x_{j}^{l}=f\left(x_{k}\cdot \omega_{j}^{l}+b_{j}^{l}\right),$$

where $x_{k}$ represents the input raw signal vector; j represents the three different convolutional scales $\left(j=1,2,3\right)$, corresponding to low, mid, and high filter sizes; $\omega_{j}^{l}$ represents the convolutional operation between the input vector and output feature map l at different scales; $b_{j}^{l}$ is a bias that corresponds to the output vector $x_{j}^{l}$, which represent the convolution operation result of the feature map l in scale j; and f is an activation function.

Then, each of the three different output vectors were subsampled by the max pooling layer in turn such that vectors of different sizes were rescaled to the same size.

### 2.2. Temporal Attention Mechanism

The acoustic signals obtained in one working condition may not follow the same temporal structure in another working condition and those signals are often masked by noises that are generated from the gearbox parts and transmitted via an elastic medium, i.e., through the air. We designed a novel temporal attention mechanism that puts more attention on the relevant information frames and suppresses noise ones to provide acoustic-based fault diagnosis under different working conditions to overcome those limitations.

**Figure 3:**
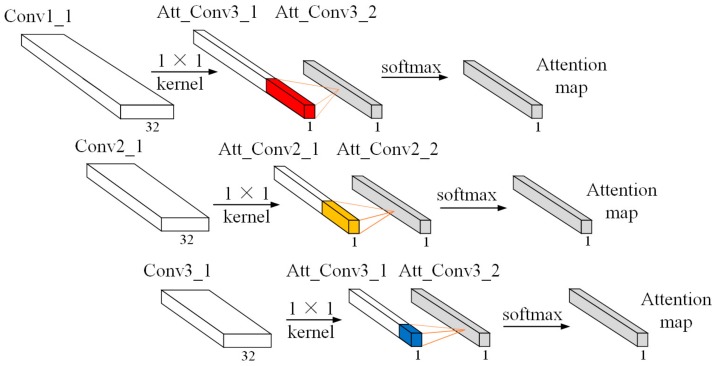
Temporal attention mechanism.

In order to reduce the impact of channel information, we first used a 1×1 kernel size with one channel to aggregate the feature maps along the channel dimension to produce a multi-scale convolutional learning structure. Then, we adopted different convolutional operations for multi-scale vectors to transform the features map into the same scale and generate a temporal attention map through the softmax activation function. Finally, we multiplied the attention map with the feature vector of the pooling layer to obtain the attention output matrix. The detailed structure of the temporal attention mechanism is shown in Figure 3 and detailed information about the operation process are given below.

Let $Convi\_1\left(i=1,2,3\right)$ denote the feature vector of the multi-scale convolution learning structure. We first operate a 1×1 kernel size over $Convi\_1\in R^{T\times 1\times C}$ to generate one channel feature map $Att\_Convi\_1\in R^{T\times 1\times 1}$. Then, multi-scale 2-D convolution with a different kernel size was adopted to learn the hidden representation and compress the features map into the same scale using different strides. The softmax activation function was applied to normalize the attention weight of Convi_1 and produce the temporal attention map. The mathematical equations are expressed as:

(2) $$Att\_Convi\_1=Conv^{1\times 1}\left(Convi\_1\right)\;i=1,2,3,$$

(3) $$Att\_Convi\_2=Att\_Convi\_1\cdot \omega_{i}+b_{i}\;i=1,2,3,$$

(4) $$A_{tm}=softmax\left(Att\_Convi\_2\right)\;i=1,2,3.$$

Finally, by multiplying the attention map $A_{tm}$ with the feature vector of the pooling layer, we obtained the attention output matrix $A_{to}$. The equation is defined as followed:

(5) $$A_{ot}^{i}={\left(x_{j}^{l}\right)}_{pool}\times A_{tm}^{i},$$

where ${\left(x_{j}^{l}\right)}_{pool}$ represents the lth feature map of the jth pooling layer (j=1,2,3). The j value refers to what is described in Section 2.1.

### 2.3. Fault Pattern Recognition Based on a CNN

We adopted a stacked convolutional neural network as a base structure for the recognition of gear fault patterns under different working conditions. The network consisted of four functional layers: the convolutional layer, the batch normalization layer (BN), the pooling layer, and the fully connected layer.

The attention output matrix from three different scales were concatenated along the channel dimension as a multi-dimensional matrix input into the convolutional layer. The stacked convolutional layer can be viewed as a fault pattern recognition module in an attention-based multi-scale CNN model. Through repetitive convolution operations, the network has the ability to learn high-level representation from the inputted multi-dimensional matrix. The process can be expressed as follows:

(6) $$y_{j}^{l}=\sum_{i=t}^{T}y_{i}^{l-1}\cdot \omega_{ij}^{l}+b_{j}^{l},$$

where $y_{i}^{l-1}$ represents the ith output feature map of the former convolutional layer, and $\omega_{ij}^{l}$ represents the convolutional kernel, which is used to operate between the ith feature map of the former layer and the jth feature map of layer l. T represents the feature atlas of the former layer and $b_{j}^{l}$ represents the bias of layer l corresponding to the output matrix $y_{i}^{l}$, which represents the convolution operation result of the jth feature map in layer l.

The output matrix of the convolutional layer is normalized by the BN layer such that the mean and variance of the feature become 0 and 1, respectively. Then, we used functions to transform and reconstruct a certain level of features to maintain the data distribution. Those equations can be expressed as:

(7) $$y_{2}=\frac{y_{1}-\mu }{\sqrt{\sigma^{2}+\varepsilon }},$$

(8) $$y_{3}=f(\gamma y_{2}+\beta ),$$

where μ and $\sigma^{2}$ represent the mean and variance of the mini-batch in Equation (7), and γ and β are the two parameters in Equation (8), which can be learned by the training network. f represents the activation function, which is used to analyze nonlinear information for the output features of the BN layer.

Then, the max pooling layer was applied to the subsample feature information of the BN layer to prevent overfitting.

Finally, through a combination of these high-level representations in a nonlinear way, a fully connected layer that recognizes gear fault patterns under different working condition was produced. The mathematical equations of the fully connected layer can be expressed as:

(9) $$h\left(y^{l}\right)=f\left(wy^{l-1}+b\right),$$

where $y^{l-1}$ represents high-level information of the former layer, $h\left(y^{l}\right)$ represents the output nonlinear information from the fully connected layer l, and ω and b represent the weight and bias, respectively.

### 2.4. Architecture and Parameters of the Attention-Based Multi-Scale CNN Model

**Figure 4:**
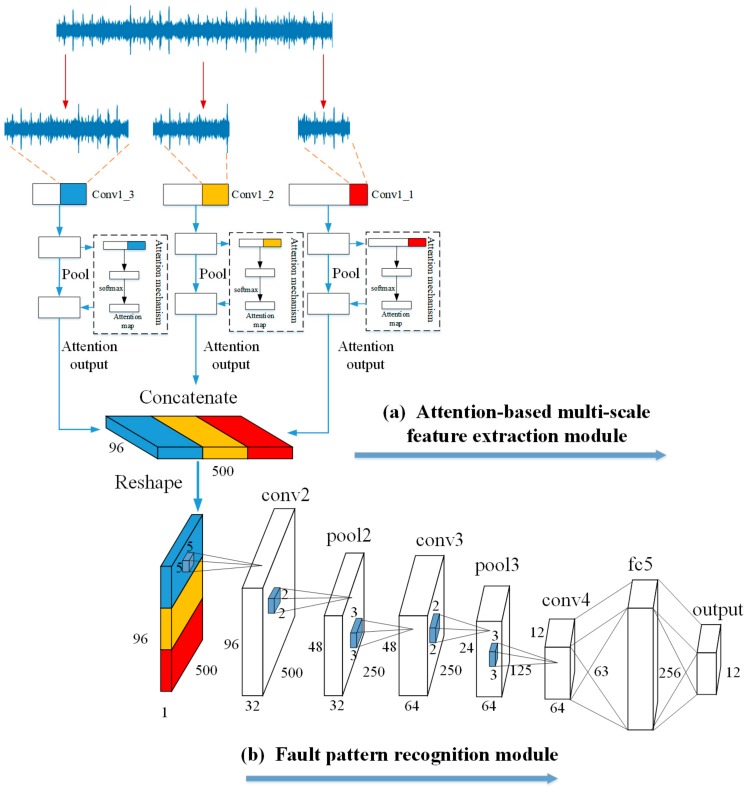
Architecture of the attention-based multi-scale convolutional neural network (CNN) model. It contains (**a**) an attention-based multi-scale feature extraction module and (**b**) a fault pattern recognition module.

The detailed information of the architecture and parameters of the attention-based multi-scale CNN model are shown in Figure 4 and Table 1, respectively. The architecture of the attention-based multi-scale CNN model consists of two parts: (a) the attention-based multi-scale feature extraction module and (b) the fault pattern recognition module.

These two modules can be divided from the concatenate layer. The attention-based multi-scale feature extraction module contains a multi-scale convolutional learning structure with an attention mechanism. The network operates on an input acoustic signal, which consists of 16,000 sampling points using three different scale convolutional neural networks of different scales. The three chosen scales are low-scale (size 11, stride1), mid-scale (size 51, stride 4), and high-scale (size 101, stride 8). Each scale has 32 filters in their convolutional layer. As an independent part in the network, the attention mechanism contains two convolutional layers to adaptively pay more attention to the relevant information frames from the output of the three convolutional layers. The kernel size of the two convolutional layers were set as (1, 1) and (64, 1) in the low scale, (1, 1) and (32, 1) in the middle scale, and (1, 1) and (16, 1) in the high scale. Then, the attention output matrix is concatenated along the channel dimension as a multi-dimensional matrix 500×1×96 (two-dimensional feature matrix × channels). Finally, in order to convolve the feature matrix from time and frequency in module (b), the attention output matrix is reshaped from 500×1×96 to 500×96×1 before being used as an input to “conv2” for further processing.

The fault pattern recognition module contains three convolutional layers, two pooling layers, and one fully connected layer. The first convolutional layer uses 32 filters with a (5,5) kernel size and stride of (1,1). The second and third convolutional layers repeatedly use 64 filters with a (3,3) kernel size and the strides were set to (1,1) and (2,2), respectively. The pooling layer is applied to subsample feature information after the first and second convolutional layers. All the kernel sizes and strides for the pooling layer were set to (2,2). In addition, the BN layer is used as a function layer, which can alleviate the problem of exploding and vanishing gradients following each convolutional layer (before the pooling layer). Finally, the output feature of the BN layer is flattened and passed to the fully connected layer with 256 nodes and the softmax activated output layer for final recognition.

## 3. Experimental Setup and Datasets

### 3.1. Experimental System

**Figure 5:**
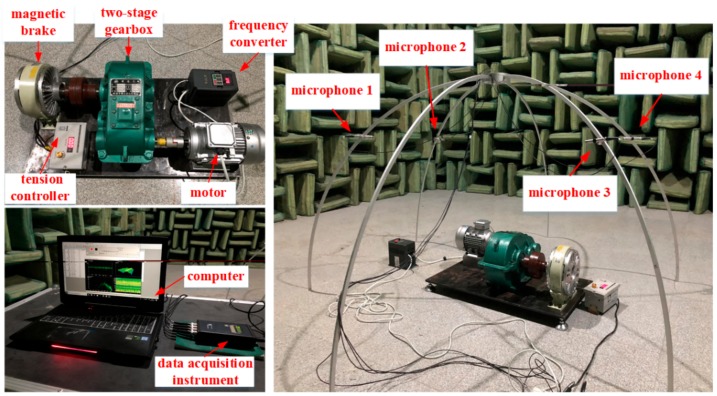
Experimental system in a semi-anechoic chamber.

Our study was a preliminary attempt at going from the theory and simulation experiment to the practical engineering application. In order to obtain pure acoustic signals that were not disturbed by environmental noise, the gear fault diagnosis experiments were conducted in a semi-anechoic chamber. The experimental system that we designed can be divided into three parts: the experiment table, the measuring system, and the data recoding software. The experiment table, as shown in the top-left corner of Figure 5 [34], consisted of the following equipment: a variable frequency motor, a two-stage gearbox, a tension controller, a frequency converter, and a magnetic brake. By adjusting the frequency converter and the tension controller, we could control the speed of the motor and simulate the load condition of the two-stage gearbox. The measuring system, as shown in the right picture of Figure 5, consisted of four free-field 4189-A-021 model microphones from Brüel & Kjær (Copenhagen, Denmark) and a data acquisition instrument from HEAD Acoustics (Herzogenrath, Germany). In our study, the four free-field microphones were arranged to provide a four-channel microphone array, where they were arranged symmetrically with a hemispherical enveloping surface and the coordinates were set according to the ISO 3745:2003 standard to collect all the gears’ acoustic signals. Then, the microphone array and data acquisition instrument were connected using a Bayonet Nut Connector (BNC) interface for data transmission. In addition, the data was recoded using Artemis 6.0 software, which is shown in bottom-left of Figure 5.

**Figure 6:**
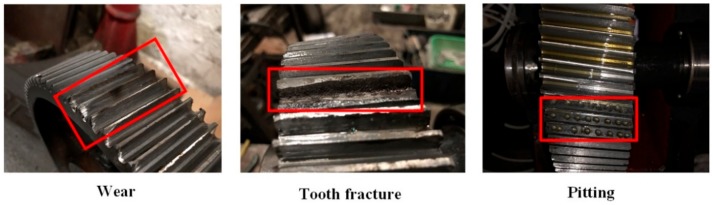
Fault pattern of the gears.

In our experiments, we chose the low-speed shaft of the two-stage gearbox as the object for detecting the gears’ fault patterns under different working conditions. The gears’ fault patterns, as shown in Figure 6 [34], consisted of a tooth fracture, pitting, and wear. We set the motor at three speeds—900 rev/min, 1800 rev/min, and 2700 rev/min—by controlling the frequency and adjusting the magnetic brake using two load conditions—0 Nm and 13.5 Nm—via tension control to simulate different working conditions. Regarding those conditions, we believe that the acoustic signal that we obtained can be viewed as only containing the gears due to the general assumption that the interference of other parts of the gearbox, such as the bearing and shaft via vibration, was minor. All the acoustic signals of the gears were recorded as an audio file that was 60 s long for further analysis. 

### 3.2. Dataset

In order to verify that the method we proposed is effective and feasible under different working conditions, we built two different datasets, A and B, that represented the two load conditions of 0 Nm and 13.5 Nm, respectively. Each dataset contained an audio file of four types of gears (one normal type and three fault types) at three different speeds, and each type was recorded to produce a four-channel audio file that was 60 s long. Each file was divided into 1-s samples with no overlap because 1-s samples are an optimal size for analysis based on empirical experiments in audio processing tasks. Then, each dataset contained 21,600 samples with 18,000 used as training samples and 3600 used as testing samples.

### 3.3. Implementation Detail

We used the cross-entropy as a loss function to train the attention-based multi-scale CNN model for multi-fault type classification under different working conditions. We applied the Adam algorithm in the training step to optimize the model, where learning rate was set to 0.003. In addition, we used rectified linear units as activation functions for each layer. When training, the dropout layer, which was followed by the fully connected layer, was employed to prevent overfitting with a 0.5 dropout rate. Finally, the early stopping approach and the no-improvement-in-10-epochs strategy was adopted to identify the number of epochs via the testing set.

## 4. Experimental Result and Analysis

### 4.1. Time and Frequency Analysis in Different Working Conditions

**Figure 7:**
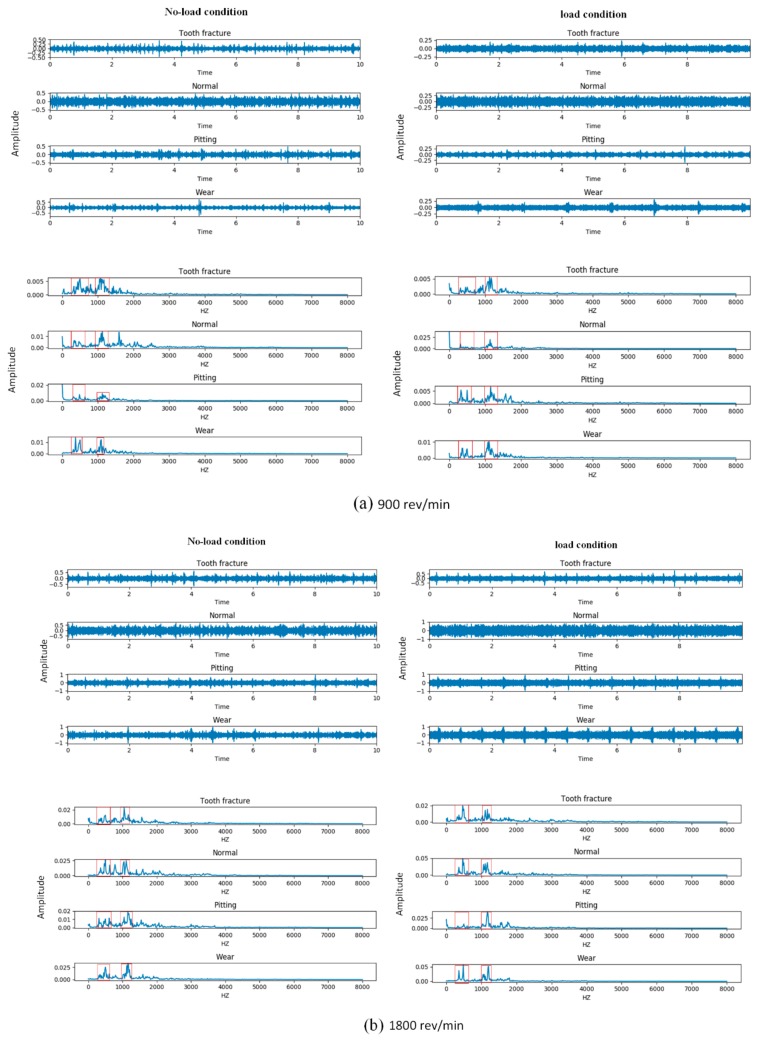

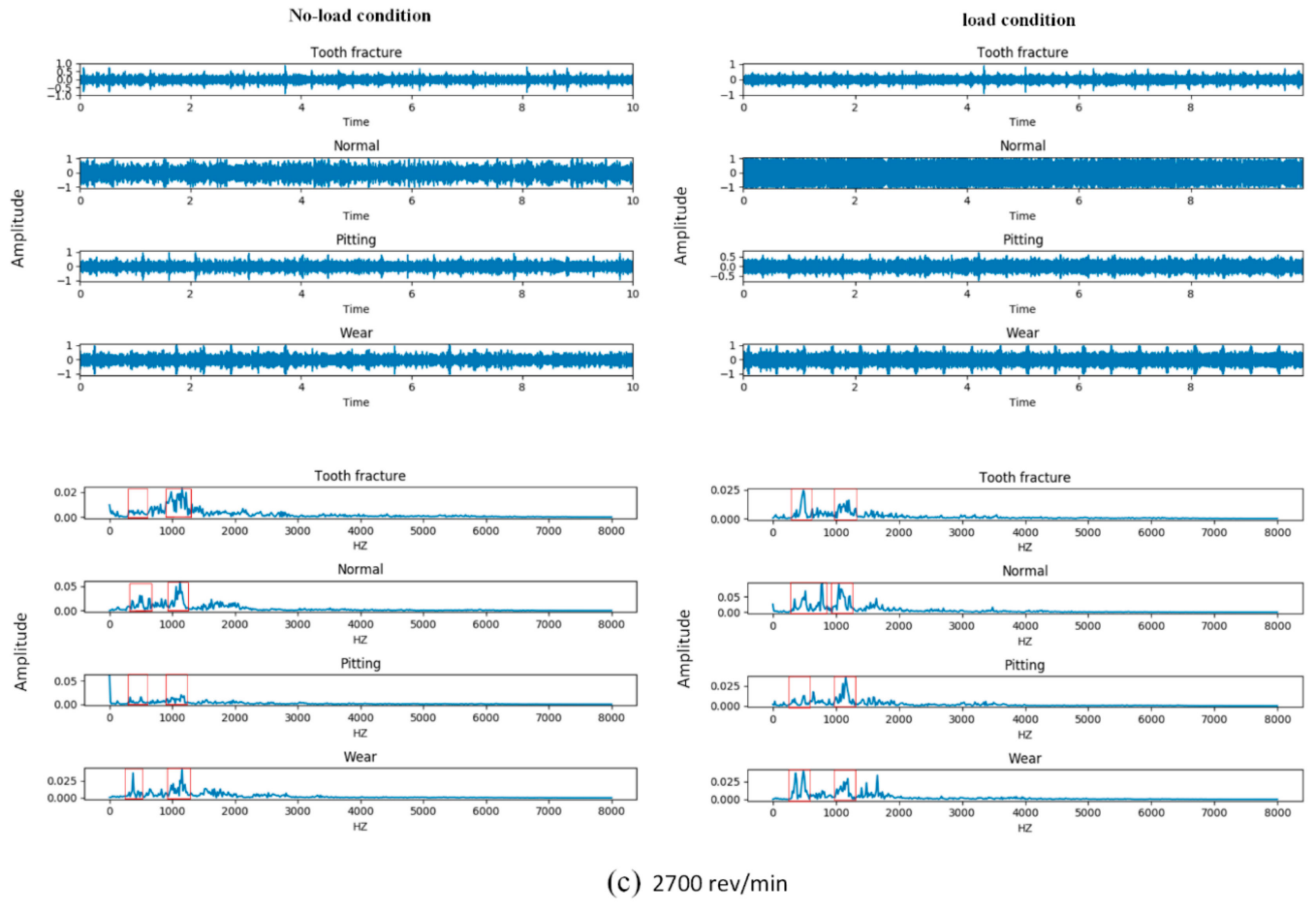
Four different types of gear signals in the time and frequency domains under two load conditions: (**a**) signal at 900 rev/min, (**b**) signal at 1800 rev/min, and (**c**) signal at 2700 rev/min.

The time and frequency domain information of the acoustic signals that we obtained from four types of gears at three different speeds under two load conditions are shown in Figure 7. The subplots (a), (b), and (c) represent the time and frequency domain signal of gears under two load conditions at 900 rev/min, 1800 rev/min, and 2700 rev/min, respectively. The left panel of each subplot shows the four types of gears in a no-load condition and the right panel shows the same type in a 13.5-Nm-load condition.

Comparing the time and frequency domain signals from the subplots, we can see that the signal amplitudes of the gears were different in the time and frequency domains under variable speed conditions. To be specific, with the increasing of the operation speed, the maximum amplitude of the majority types of gears in the time domain also increased under the same load condition. Meanwhile, from the frequency domain signal, we found that the magnitude of the frequency amplitude for the same types of fault signals under variable speed conditions was different, but the distribution of the amplitude was not affected by the varying speed. For example, in the frequency domain, the normal gears had a higher amplitude at 2700 rev/min speed condition than the same type of gears at 900 rev/min and 1800 rev/min under the no-load condition, but the distribution of the amplitude, which was concentrated in the range of 0–500 Hz and 1000–1200 Hz, was consistent in the three different speed conditions. Based on the description above, we could infer that the variable speed caused the amplitude modulation phenomenon of the acoustic signal, but did not influence the frequency modulation.

Furthermore, comparing the time and frequency domain signals under the no-load condition and load condition at 900 rev/min, 1800 rev/min, and 2700 rev/min, we observed that the acoustic signals for four types of gears seemed to be obviously different in the two load conditions. From the time-domain signal, we saw that the gears with a wear type and the gears with pitting under the no-load conditions had a higher amplitude range than the same fault type under the 13.5-Nm-load condition at 900 rev/min, but it was the opposite at 1800 rev/min. Moreover, the waveform of each type of gear in the time domain were different, which means the temporal structure and energy modulation patterns of the signal under the two load conditions were diverse. Meanwhile, according to the magnitude and distribution of the frequency amplitude in the ranges of 0–500 Hz and 1000–1200 Hz, we found that the frequency signal of the four types of gears under the two loads were also different, especially for the normal and pitting types of gears. As for the normal gears at 900 rev/min, the frequency component under the no-load condition was around 0–500 Hz and 1000–1200 Hz, while the frequency component under the load condition was around 1000–1200 Hz. Equally, the gears with the pitting type had the same phenomenon at 1800 rev/min. This indicates that the acoustic signals of gears under different load conditions were not only affected by the amplitude modulation, but also by the frequency modulation. In addition, another interesting phenomenon we observed was that the type of gear signal that we obtained under the no-load condition at one speed may follow the same magnitude and distribution of frequency amplitude under the load condition at another speed. For example, the normal gears under the no-load condition at 900 rev/min had a similar amplitude and distribution of frequency to the same gear type under the 13.5-Nm-load condition at 1800 rev/min. This means that the gear fault diagnosis under variable load conditions is more complex and difficult than that of a variable speed condition. Therefore, we proposed an attention-based multi-scale CNN model for gear fault diagnosis under variable load conditions.

### 4.2. Effectiveness of the Multi-Scale Convolution Operation

In order to verify the hypothesis that the multi-scale convolutional learning structure is superior to the single-scale convolutional structure in for gear fault diagnosis tasks, we first compared the performance of our multi-scale convolutional neural network with that of a low-scale convolutional neural network, mid-scale convolutional neural network, and high-scale convolutional neural network with no attention mechanism. These three models remained at only one scale each, which is shown in Figure 2. The input and the rest of the network structure is the same as in Figure 4 for fair comparison. The two evaluation methods were designed to evaluate the performance of models under different load conditions:

In evaluation A, we used the training samples of dataset A to train these CNN models and test it on the test samples of dataset B.

In evaluation B, we used the training samples of dataset B to train these CNN models and test it on the test samples of dataset A.

The classification accuracy was used as the evaluation criterion. The equation is defined as:

(10) $$ACC=\frac{N_{1}}{N_{2}},$$

where $N_{1}$ represents the number of test samples that were predicted properly and $N_{2}$ represents the total number of test samples.

The test results of the multi-scale network and single-scale network in the two evaluation methods are shown in Table 2. From the result, we saw that the recognition accuracy of the multi-convolutional neural network reached 81.1% and 71.0% for the two evaluation methods, respectively. This was an improvement of 2.5%, 2.3%, and 1.6% compared with the low-scale, mid-scale, and high-scale networks for evaluation A, respectively. Also, the multi-scale convolutional neural network achieved the best accuracy for evaluation B. The improvement from the single-scale model to the multi-scale model proved that the model, which contained different scales, was capable of learning more discriminative features from the waveforms.

**Figure 8:**
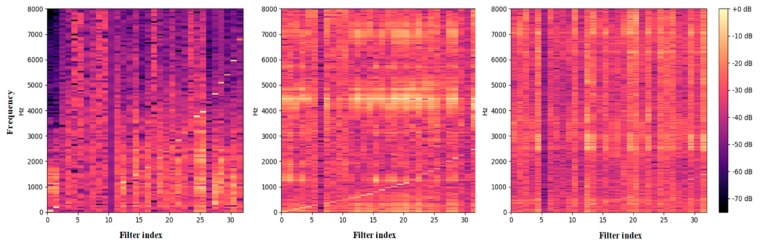
Frequency magnitude response of the multi-scale convolutional filters of the first layer. The filters are sorted by their center frequency. Left shows the frequency response of the low-scale network, middle shows the frequency response of the mid-scale network, and right shows the frequency response of the high-scale network.

To further understand how the multi-scale convolution operations help to improve the performance of fault pattern recognition, we visualized the frequency magnitude of the response of the multi-scale feature maps Conv1 of the model in Figure 8. As indicated in this figure, the 32 filters were viewed as band-pass filters to learn a particular frequency area, and each filter was sorted based on their center frequencies. From the left picture of Figure 8, we observed that the curve of the center frequency almost matched the sound feature of the human auditory system. This means that the low-scale structure was able to extract features from all frequency areas. Conversely, the high-scale structure, which is shown in the right of Figure 8, was located in the low-frequency area with fine-grained filters. It shows that the high-scale structure tended to concentrate on low-frequency components and ignore high-frequency information. Moreover, the mid-scale performed between the low-scale and high-scale networks. In general, a model with a narrow kernel size can cover all frequency areas but obtains a low-frequency resolution, and a model with a wide kernel size does not have sufficient filters in the high-frequency range but gives good frequency resolution. It indicates that learning structures of different scales can extract discrepant features based on what they can efficiently represent. This may explain the result that we present in Table 2 where the multi-scale models obtain a better performance than the single-scale models. 

### 4.3. Comparison of a Standard CNN Model and Attention Models 

To verify the effectiveness of the attention mechanism, we applied the attention mechanism to a multi-scale convolutional neural network and each single-scale network for a fair comparison. The performances of the models are summarized in Table 3. 

In Table 3, it is shown that the performances of each model presented a significant improvement when using an attention mechanism compared with the standard model, which did not use an attention mechanism, especially in the case of the multi-scale convolutional model. The attention-based multi-scale CNN model achieved a 93.3% accuracy in evaluation A and 82.8% in evaluation B. This was 12.2% and 11.8% higher than the accuracy of the standard multi-scale CNN model. Meanwhile, by using an attention mechanism, the improvement range of recognition performance of the single-scale model was from 6.8% to 8.8% for evaluation A and from 5.4% to 14.1% for evaluation B. The test results indicate that the attention mechanism was effective at gear fault diagnosis under different load conditions. 

**Figure 9:**
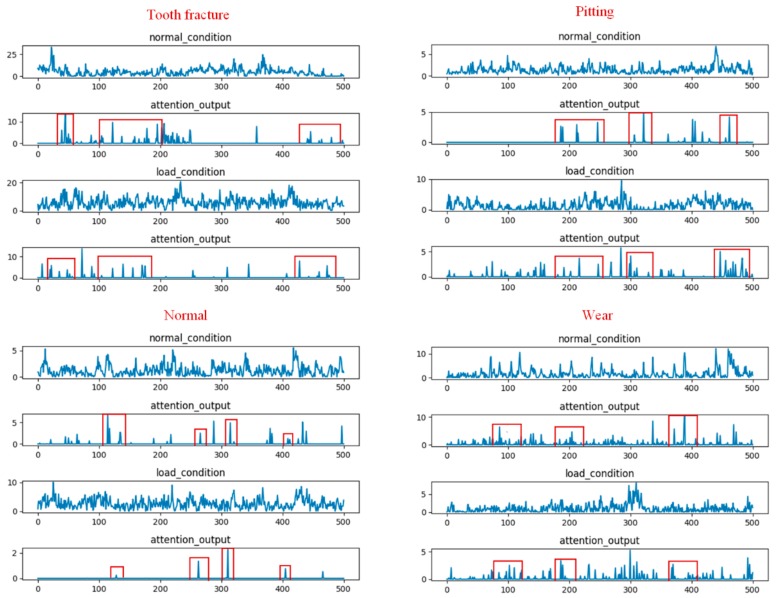
Visualization results of randomly selected filters from the multi-scale pool layer and temporal attention output for four different types of gear input signals under the two load conditions corresponding to the attention based multi-scale CNN model for evaluation A.

To provide a better understanding how a temporal attention mechanism helped to improve the performance of the multi-scale CNN model in the gear fault diagnosis task, we visualized the results of randomly selected filters from the multi-scale pool layer and temporal attention output for four different types of gear input signals under the two load conditions. Figure 9 shows the visualization result of the attention-based multi-scale CNN model for evaluation A. The first and third rows represent the waveform under the no-load condition and 13.5-Nm-load conditions, respectively. The second and fourth rows represent the attention output corresponding to the waveform of the two load conditions, respectively. From this figure, we found that the temporal attention mechanism, which was based on the multi-scale CNN model, was able to adaptively focus on the relevant temporal information from the different waveforms of the two load conditions while reducing the impact of the data distribution variation. Furthermore, from the attention output, we found that the attention weights of the four gear types were different for different time stamps. For example, the tooth fracture condition in gears had three high-weighted areas at time stamps in the ranges of #30–#50, #100–#200, and #430–#500 frames, while the high-weighted areas of the gears with pitting were in the ranges of #180–#230, #300–#320, and #450–#480. This may indicate that the learned temporal attention was able to detect the essential feature information required to distinguish these four types of gears under the two load conditions.

**Figure 10:**
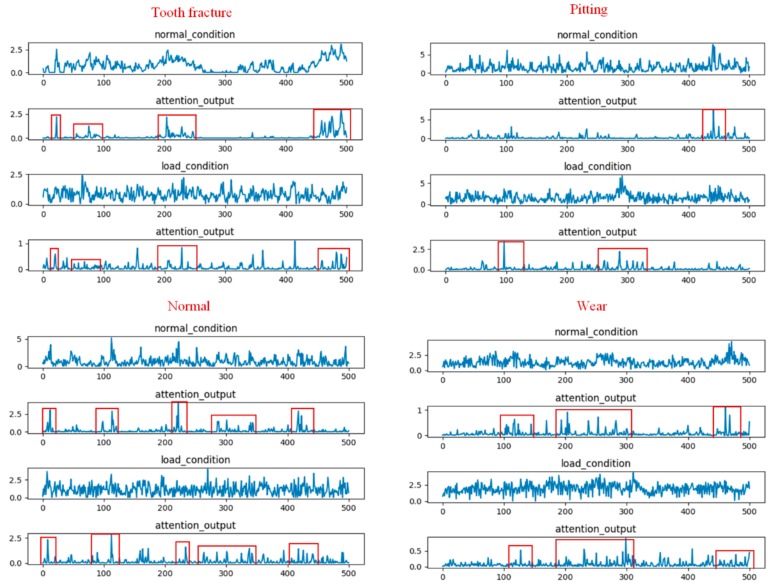
Visualization results of randomly selected filters from the multi-scale pool layer and temporal attention output for four different types of raw gear signals under the two load conditions corresponding to the attention based multi-scale CNN model for evaluation B.

Figure 10 shows the visualization results of the attention based multi-scale CNN model for evaluation B. From this figure, we found two phenomena. First, the learned temporal attention was also able to locate the meaningful temporal parts from the different waveforms of the two load conditions for evaluation B. Meanwhile, the four types of gears could be easily distinguished according to the distribution of the time stamps. This visualization result was similar to that obtained for evaluation A. Second, the high-attention-weights area of the four gear types for evaluation B were not consistent with the high-attention-weights area for evaluation A. This may be due to the fact that the temporal attention mechanism, which was trained on different datasets, could generate different attention weights to focus on different temporal parts. 

The above visualization results may explain why the temporal attention mechanism was effective at gear fault diagnosis under different load conditions.

### 4.4. Comparison between the Attention-Based Multi-Scale CNN Model and Other Methods 

In this section, we compare our model with traditional methods that combine one of the most powerful acoustic features, namely MFCC (Mel-frequency cepstral coefficients), with a convolutional neural network and several classic fault diagnosis methods, which have been successfully used in fault pattern recognition tasks based on time, frequency, manual features, and machine learning algorithms, to analyze the performance of our model. Meanwhile, the end-to-end model, which is based on time and frequency domain signals that we proposed in a previous work, was also used for further comparison. 

We adopted the commonly used parameter [35,36] to construct the MFCC feature, the first derivative of the MFCC feature (MFCC-delta) and the second derivative of the MFCC feature (MFCC-delta-delta) as matrix features. Then, we fed it into the convolutional neural network, whose structure is the same as module (b) of the multi-scale CNN model in Figure 3 to provide a fair comparison. The procedure of the classic fault diagnosis methods can be divided into two parts: manual feature extraction and fault identification. The manual features that we extracted include the root-mean-square error (RMS) [37], spectral centroid [38], and Mel spectrogram in the log domain [39], which represent the popularity acoustic features in the time, frequency, and time–frequency domains, respectively. We fused these manual features at the feature level for improving the representation of the fault information. Then, we fed it into several classic machine learning classifiers [40,41,42,43,44,45] that are widely used in fault diagnosis tasks for comparison. The end-to-end model that we proposed in a previous work [34] used time and frequency signals as raw input signals to detect the gear fault patterns. The test results of those methods on two datasets are shown in Table 4.

From the results, we found that the accuracy of the MFCC-delta CNN and the MFCC-delta-delta CNN had similar performances, which were better than the MFCC CNN, but the accuracy of the best traditional method, namely the MFCC-delta CNN, was 10% lower than our attention-based multi-scale CNN for evaluation A. Moreover, the prediction accuracy of all methods declined for evaluation B, but those traditional methods gave worse performances compared with our model. The above conclusions indicate that our attention-based multi-scale CNN structure could learn more discriminative features than traditional manual features by combining attention-based multi-scale information.

Furthermore, the recognition accuracy of our attention multi-scale CNN model reached 93.3% for evaluation A and 82.8% for evaluation B. This was 5.4 % and 6.0% higher than the accuracy of the best classic diagnosis method, namely manual features + k-nearest neighbor (KNN).

In addition, our attention multi-scale CNN model showed an improvement of 3.6% when compared with the end-to-end CNN model for evaluation A. Furthermore, our model achieved at least a 1.7% improvement over the end-to-end CNN model for evaluation B. The improvement proved that the multi-scale CNN model based on the attention mechanism was able to adaptively learn more efficient frequency representations using attention based multi-scale band-pass filters from raw waveforms without frequency input signals. 

**Figure 11:**
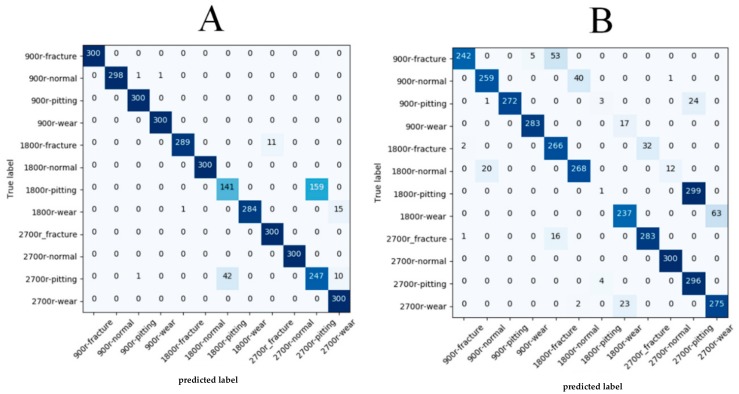
Confusion matrixes for the proposed attention-based multi-scale CNN model. The left matrix shows the statistics for evaluation (**A**), while the right matrix shows the statistics for evaluation (**B**).

In Figure 11, we provide the confusion matrix in order to further analyze the performance of our proposed method regarding the two evaluation methods. From the confusion matrix for evaluation A, we observed that most fault patterns under different working conditions could obtain a high classification accuracy, except for gears with pitting types at 1800 rev/min and 2700 rev/min. The two categories appeared to be easily misclassified due to the signal of pitting types at 1800 rev/min under the no load condition being similar to the same type at 2700 rev/min under the 13.5-Nm-load condition, as caused by the amplitude modulation and frequency modulation, the phenomena of which is discussed in Section 4.1. Furthermore, from the confusion matrix for evaluation B, we noticed that the recognition accuracy declined for some classes, especially for gears with pitting types at 1800 rev/min. According to Figure 10 in Section 4.3, we found that the temporal parts, where the attention mechanism was located, were totally different for pitting-type gears, which means that the discriminative feature of pitting-type gears that the model learned were different at 1800 rev/min under the two load conditions. This may explain why the pitting-type gears displayed a lower performance for evaluation B. As for the rest of the classes, we suspect that the easy misclassification was due to some extra information between the acoustic signals and fault types existing in the load working condition but not in the no-load working condition such that the training the model in the load condition and testing it in no-load condition could be viewed as diagnosis fault patterns with noise, which led to some degree of overfitting in our model. In general, the attention-based multi-scale CNN model that we propose still had better generalization capabilities under variable load conditions compared with the other methods.

**Figure 12:**
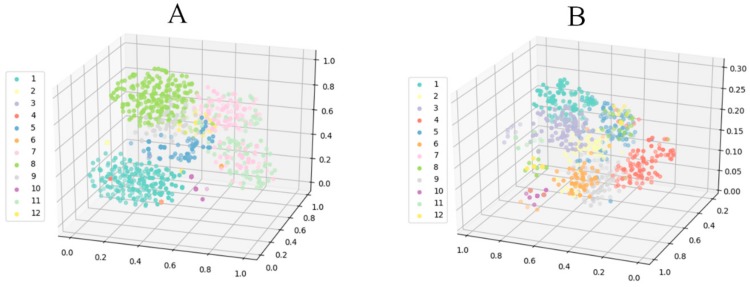
Feature visualization via t-SNE (t-distributed stochastic neighbor embedding). Left shows the feature representations for the last fully connected layer of attention-based multi-scale CNN model for evaluation (**A**). Right shows the feature representations for evaluation (**B**).

Finally, to better show the performance of the attention-based multi-scale CNN model for the two evaluation methods, we visualized the prediction result of the model by using a t-SNE (t-distributed stochastic neighbor embedding) algorithm (Figure 12). The t-SNE algorithm was operated on the output matrix of the last fully connected layer to reduce the dimensionality to conveniently show the classification result in three-dimensional space. From these visual results of three-dimensional space, we observed that most features clustered successfully around the two evaluation methods, which also proved that our attention-based multi-scale model achieved a better performance at acoustic signal-based gear fault diagnosis under different working conditions.

## 5. Conclusions

In this paper, a novel ABD method was proposed for gear fault diagnosis under different working conditions based on a multi-scale convolutional learning structure and attention mechanism. By using a multi-scale convolutional learning structure, our model was able to automatically mine more efficient feature representations from raw acoustic signals. It achieved better performance than the single-scale models. Based on the multi-scale convolutional learning structure, a novel attention mechanism, which operated on the convolutional layer, was applied to adaptively extract relevant fault information and reduce the data distribution variation under different working conditions. The experimental result for the two evaluation methods showed that the accuracy of our model reached 93.3% and 82.8%, respectively. All the performance metrics were higher than those of the standard CNN model, end-to-end CNN model based on time and frequency domain signals, and other traditional fault diagnosis methods with manual features. This indicates that our model was more effective at acoustic-based gear fault diagnosis under different working conditions. Furthermore, we analyzed the discrimination of different scale convolutional learning structures using feature representations and visualized the attention output to provide insight into the reason for the performance improvement in the gear fault diagnosis task. In future, the attention-based mechanism method can be further developed in the ABD field. We will continue to explore the effectiveness of the attention-based method for bearing fault diagnosis, the multi-fault diagnosis of gears, and the coupling fault diagnosis of gearboxes. Meanwhile, we will extend our attention-based method into other fault diagnosis fields that are out of the controlled environment, such as fault diagnosis in normal environmental conditions and strong environmental noise conditions.

## Figures and Tables

**Table 1:** Parameters of the attention-based multi-scale CNN model.

Layer	Input Shape	Filter	Kernel Size	Stride	Output Shape
Conv1_1	[batch,16000,1,4]	32	(11,1)	(1,1)	[batch,16000,1,32]
Conv2_1	[batch,16000,1,4]	32	(51,1)	(4,1)	[batch,4000,1,32]
Conv3_1	[batch,16000,1,4]	32	(101,1)	(8,1)	[batch,2000,1,32]
Conv2	[batch,500,96,1]	32	(5,5)	(1,1)	[batch,500,96,32]
Pool2	[batch,500,96,32]	-	(2,2)	(2,2)	[batch,250,48,32]
Conv3	[batch,250,48,32]	64	(3,3)	(1,1)	[batch,250,48,64]
Pool3	[batch,250,48,64]	-	(2,2)	(2,2)	[batch,125,24,64]
Conv4	[batch,125,24,64]	64	(3,3)	(2,2)	[batch,63,12,64]
Fc5	[batch,48384]	-	-	-	[batch,256]
Output	[batch,256]	-	-	-	[batch,12]

**Table 2:** Prediction accuracy of multi-scale CNN and single CNN model.

CNN Model without Attention	Accuracy (%)	
	Evaluation A Train on A Test on B	Evaluation B Train on B Test on A
**Multi-scale**	**81.1**	**71.0**
Low-scale	78.6	70.8
Mid-scale	78.8	64.4
High-scale	79.5	62.3

**Table 3:** Prediction accuracy when applying an attention mechanism.

CNN Model With Attention	Accuracy (%)	
	Evaluation A Train on A Test on B	Evaluation B Train on B Test on A
**Multi-scale**	**93.3**	**82.8**
Low-scale	86.7	76.2
Mid-scale	87.6	75.1
High-scale	86.3	76.4

**Table 4:** Prediction accuracy comparison of our attention-based multi-scale CNN model and other methods. GBDT: Gradient Boosting Decision Tree, KNN: k-nearest neighbor, SVM: support vector machine.

Method	Feature	Recognition Model	Accuracy (%)	
			Evaluation A Train on A Test on B	Evaluation B Train on B Test on A
**Attention-based multi-scale CNN**	**Time signal**	**Multi-scale CNN**	**93.3**	**82.8**
MFCC CNN	MFCC	(b) module	78.7	59.4
MFCC-delta CNN	MFCC-delta	(b) module	83.3	58.8
MFCC-delta-delta CNN	delta-Deltas	(b) module	82.6	57.4
End-to-end stacked CNN	Time–frequency signal	_	89.7	81.1
Multiple feature + KNN	Multiple feature	KNN	87.9	76.8
Multiple feature + SVM	Multiple feature	SVM	83.2	66.7
Multiple feature + GBDT	Multiple feature	GBDT	71.5	48.4
